# Muscle damaging eccentric exercise attenuates disuse-induced declines in daily myofibrillar protein synthesis and transiently prevents muscle atrophy in healthy men

**DOI:** 10.1152/ajpendo.00294.2021

**Published:** 2021-10-11

**Authors:** Tom S. O. Jameson, Sean P. Kilroe, Jonathan Fulford, Doaa R. Abdelrahman, Andrew J. Murton, Marlou L. Dirks, Francis B. Stephens, Benjamin T. Wall

**Affiliations:** ^1^Nutritional Physiology Group, Department of Sport and Health Sciences, College of Life and Environmental Sciences, University of Exeter, Exeter, United Kingdom; ^2^Peninsula NIHR Clinical Research Facility, College of Medicine and Health, University of Exeter, Exeter, United Kingdom; ^3^Department of Surgery, University of Texas Medical Branch, Galveston, Texas; ^4^Department of Nutrition and Metabolism, Center for Recovery, Physical Activity and Nutrition, University of Texas Medical Branch, Galveston, Texas; ^5^Sealy Center of Aging, University of Texas Medical Branch, Galveston, Texas

**Keywords:** deuterated water, immobilization, muscle damage, muscle disuse, muscle protein synthesis

## Abstract

Short-term disuse leads to muscle loss driven by lowered daily myofibrillar protein synthesis (MyoPS). However, disuse commonly results from muscle damage, and its influence on muscle deconditioning during disuse is unknown. Twenty-one males [20 ± 1 yr, BMI = 24 ± 1 kg·m^−2^ (± SE)] underwent 7 days of unilateral leg immobilization immediately preceded by 300 bilateral, maximal, muscle-damaging eccentric quadriceps contractions (DAM; subjects *n* = 10) or no exercise (CON; subjects *n* = 11). Participants ingested deuterated water and underwent temporal bilateral thigh MRI scans and vastus lateralis muscle biopsies of immobilized (IMM) and nonimmobilized (N-IMM) legs. N-IMM quadriceps muscle volume remained unchanged throughout both groups. IMM quadriceps muscle volume declined after 2 days by 1.7 ± 0.5% in CON (*P* = 0.031; and by 1.3 ± 0.6% when corrected to N-IMM; *P* = 0.06) but did not change in DAM, and declined equivalently in CON [by 6.4 ± 1.1% (5.0 ± 1.6% when corrected to N-IMM)] and DAM [by 2.6 ± 1.8% (4.0 ± 1.9% when corrected to N-IMM)] after 7 days. Immobilization began to decrease MyoPS compared with N-IMM in both groups after 2 days (*P* = 0.109), albeit with higher MyoPS rates in DAM compared with CON (*P* = 0.035). Frank suppression of MyoPS was observed between *days 2* and *7* in CON (IMM = 1.04 ± 0.12, N-IMM = 1.86 ± 0.10%·day^−1^; *P* = 0.002) but not DAM (IMM = 1.49 ± 0.29, N-IMM = 1.90 ± 0.30%·day^−1^; *P* > 0.05). Declines in MyoPS and quadriceps volume after 7 days correlated positively in CON (*r*^2^ = 0.403; *P* = 0.035) but negatively in DAM (*r*^2^ = 0.483; *P* = 0.037). Quadriceps strength declined following immobilization in both groups, but to a greater extent in DAM. Prior muscle-damaging eccentric exercise increases MyoPS and prevents loss of quadriceps muscle volume after 2 (but not 7) days of disuse.

**NEW & NOTEWORTHY** We investigated the impact of prior muscle-damaging eccentric exercise on disuse-induced muscle deconditioning. Two and 7 days of muscle disuse per se lowered quadriceps muscle volume in association with lowered daily myofibrillar protein synthesis (MyoPS). Prior eccentric exercise prevented the decline in muscle volume after 2 days and attenuated the decline in MyoPS after 2 and 7 days. These data indicate eccentric exercise increases MyoPS and transiently prevents quadriceps muscle atrophy during muscle disuse.

## Graphical abstract

**Figure:**
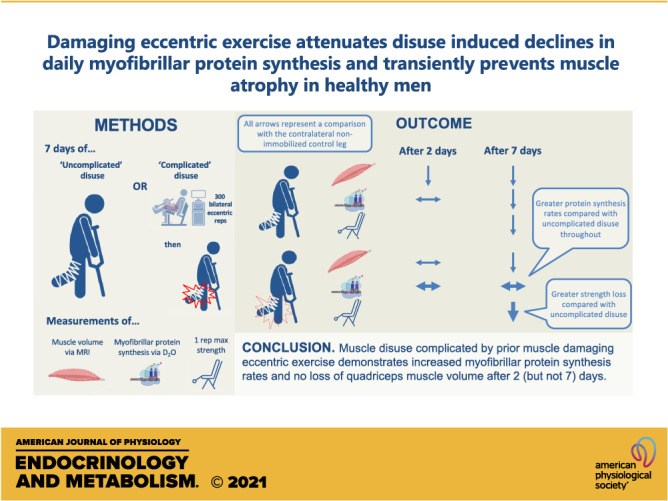

## INTRODUCTION

A short-term period of rehabilitative physical inactivity or complete limb immobilization is often prescribed following acute musculoskeletal injury (e.g., sports injuries) to facilitate healing and prevent further injury (1). Experimental physical inactivity is commonly modeled in the laboratory by subjecting healthy, uninjured individuals to acute (e.g., 1–14 days) muscle disuse via limb immobilization (e.g., see Refs. 2–14). Using such models of “uncomplicated” (i.e., removal of contractile activity only) muscle disuse, we (6, 7, 15–17) and others (4, 5, 8–14) have measured rapid [within 2 days (15)] and substantial [approx. 270 g tissue/wk (15)] muscle atrophy, which is greatest within the first 14 days (18) and accompanied by associated and disproportionately high declines in strength (2, 8, 10–16) and metabolic function (19, 20).

Mechanistically, uncomplicated human muscle disuse atrophy can largely be explained by declines in myofibrillar protein synthesis (MyoPS) rates. In support, limb immobilization of healthy volunteers leads to reduced postabsorptive MyoPS rates and the development of resistance to the anabolic properties of dietary protein ingestion measured over several hours under controlled laboratory conditions (3, 7, 9, 21–23). Furthermore, we have recently reported that these acute metabolic perturbations translate to reduced daily MyoPS rates measured under free-living conditions during 2–7 days of limb immobilization in healthy men (16, 24), with the extent of the decline positively correlating with the loss of muscle mass (24).

A major issue with using models of uncomplicated disuse to confer a mechanistic understanding of muscle atrophy following injury or illness is they do not consider other factors likely to influence muscle deconditioning that would ordinarily be present (i.e., more “complicated” disuse). Musculoskeletal injury is typically associated with muscle damage and inflammation (25) both of which have been associated with activation of anabolic (26–28) and catabolic (29, 30) processes in skeletal muscle. We (26, 27) and others (31, 32) have previously used a single bout of high-volume and high-intensity eccentric muscle contractions to experimentally induce muscle damage, which manifests as ultrastructural myofibrillar disruption (31–34) and an associated decline in muscle contractile function within 2 days, which is typically recovered within 7 days (26, 35). Mechanistically, these studies have also demonstrated that such eccentric exercise-induced muscle damage is associated with transient inflammation in skeletal muscle and increased daily free-living MyoPS rates for up to 3 days postexercise (26, 27, 36–39). These data suggest the induction of muscle damage via eccentric muscle contractions before a period of disuse (i.e., similar to expected during injury) could have a profound effect on the subsequent rate of muscle deconditioning, which is likely to change temporally, and is so far not considered by translating laboratory findings to assertions of atrophy during injury or illness.

In the present study, we used 300 bilateral and maximal eccentric knee extensor contractions to model (more) complicated muscle disuse and test the bidirectional hypothesis that exercise-induced muscle damage would impact subsequent muscle deconditioning responses during 1 wk of unilateral leg immobilization (via a leg brace with crutches for ambulation). We applied isokinetic dynamometry to elicit quadriceps muscle damage, and magnetic resonance imaging (MRI), and an oral deuterated water stable isotope approach to assess the temporal impact of prior damage-inducing exercise on muscle mass and daily free-living MyoPS rates, respectively, compared with uncomplicated (i.e., immobilization with no prior exercise) disuse.

## METHODS

### Participants

Twenty-two young, healthy males (age = 20 ± 1 yr, BMI = 24 ± 1 kg·m^−2^) volunteered to take part in the present study. Only young males were included in the present study as both age (4) and sex (40) can influence the rate of muscle disuse atrophy, and our goal was to maintain a homogenous population to investigate physiological mechanisms within the current study. Participants attended the laboratory for a routine medical screening and completed a general medical questionnaire to assess their eligibility for participation. Exclusion criteria included: *1*) (family) history of deep vein thrombosis/cardiovascular disease, *2*) metabolic disorders, *3*) musculoskeletal/orthopedic disorders, *4*) a body mass index of > 25.8 kg·m^−2^ or < 18.5 kg·m^−2^, *5*) a musculoskeletal injury to the legs within the 12 mo preceding participation, *6*) participating in a structured resistance or endurance exercise training program within the 6 mo preceding participation, *7*) use of anticoagulants, and *8*) chronic consumption of any nutritional supplement or medication before and during the study. All subjects were informed of the nature and possible risks of the experimental procedures before providing written, informed consent. The study was approved by the Sport and Health Sciences Ethics Committee of the University of Exeter (171206/B/08) in accordance with the guidelines set out in the Declaration of Helsinki. The study was registered at ClinicalTrials.Gov (NCT03559452).

### Experimental Design

**Figure 1.**
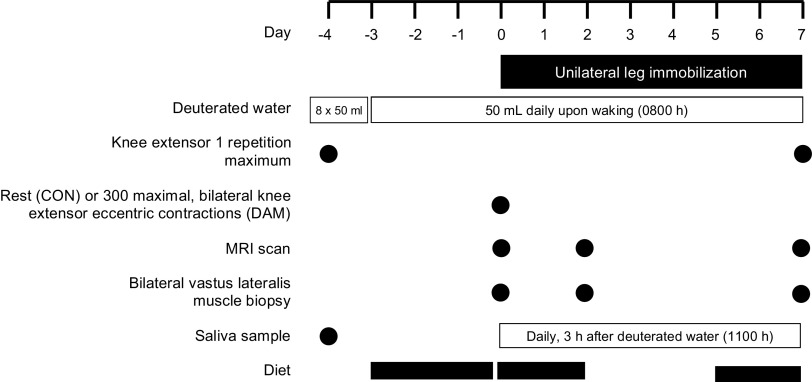
Schematic representation of the experimental protocol. Participants underwent a 7-day unilateral leg immobilization protocol which was immediately preceded by no exercise (CON) or 300 eccentric muscle damaging knee extensor contractions performed in both legs (DAM). Loading of orally ingested 70% deuterated water began at 0800 h on *day −4* with 8 × 50 mL doses consumed every 1.5 h and body water deuterium enrichment was maintained thereafter with daily 50 mL doses. Bilateral vastus lateralis muscle biopsies and thigh MRI scans were performed before (*day 0*), and after 2 and 7 days of immobilization, and knee extensor 1-repetition maximum (1-RM) was measured before and after immobilization. Saliva samples were collected daily to measure body water deuterium enrichment and diet diaries were completed 3 days before immobilization and during the first and last 2 days of immobilization.

An overview of the experimental protocol is shown in Fig. 1. Following successful completion of a screening visit and a separate familiarization visit to the eccentric contraction and one-repetition maximum protocols, participants attended the laboratory for a further four visits across an 11-day experimental period, which included a 7-day period of unilateral leg immobilization. In a parallel groups design, participants performed either no exercise (i.e., uncomplicated muscle disuse; CON; *n* = 11) or a bout of 300 bilateral and maximal eccentric muscle contractions of the knee extensors (DAM; *n* = 11) immediately before the 7-day unilateral leg immobilization period. To measure daily myofibrillar protein synthesis (MyoPS) rates throughout the immobilization period, participants underwent a deuterated water dosing protocol on *day −4* designed to achieve and maintain 0.8% – 1.0% of body water deuterium enrichment during the immobilization period in line with previous work from ourselves (16, 24) and others (41, 42). Participants arrived at the laboratory at 0800 h on *day 0* of the experimental period for the first of three experimental test days. During this visit, bilateral muscle biopsies from the m. vastus lateralis were collected, followed by a bilateral MRI scan (see *Magnetic Resonance Imaging and Muscle Volume Calculations* section below*)* of the thigh muscles. Then, half of participants (i.e., DAM) performed a bout of 300 maximal eccentric contractions in both legs, which was matched for volume and intensity between legs (see *Eccentric Exercise and 1-Repetition Maximum Testing Protocols* section below), whereas the other half of participants (i.e., CON) performed no exercise. At 0900–1000 h a leg brace was fitted to one leg (IMM; randomized for leg dominance) with the contralateral leg acting as a within-participant non-immobilized control leg (N-IMM) and the 7-day immobilization period commenced. After 2 and 7 days of immobilization, participants returned to the laboratory for further bilateral muscle biopsies and MRI scans under overnight fasted conditions (participants were transported to and from the MRI scanner in a wheelchair to ensure no contraction or weight-bearing of the immobilized leg). Knee extensor 1-repetition maximum (1-RM) was determined in both legs separately on *day −4* and *day 11* once the leg brace was removed after the 7-day immobilization period (see *Eccentric Exercise and 1-Repetition Maximum Testing Protocols* section below). Muscle biopsies were obtained under local anesthesia using the percutaneous Bergstrom needle biopsy technique (43), from the m. vastus lateralis of both legs ∼15 cm above the patella and ∼3 cm below the fascia. Muscle tissue was immediately dissected from any visible blood or nonmuscle tissue and frozen in liquid nitrogen and stored at −80°C until further analysis.

### Eccentric Exercise and 1-Repetition Maximum Testing Protocols

Participants in DAM performed eccentric contractions on a Biodex System 3 isokinetic dynamometer (Biodex Medical Systems, Shirley, NY) using a protocol previously used by ourselves (26, 27) and others (36, 44, 45) to induce muscle damage specifically of the knee extensors. Briefly, participants were seated with 85° of hip flexion and extraneous movement was restrained using the shoulder, hip, and thigh straps. Participants first performed 300 (10 sets of 30 repetitions) voluntary maximal, isokinetic, eccentric contractions of the knee extensors in the leg randomized to be subsequently immobilized, with the concentric phase being automated and participants instructed to relax. Then, during a 10-min rest period, the Biodex was reconfigured and participants performed the same 300 maximal eccentric contractions in the contralateral leg. Each contraction was performed at 60°·s^−1^ over an 80° range of motion, which ended at full voluntary knee flexion. Each set was separated by 120 s of rest. Participants were instructed to resist the eccentric movement maximally throughout the full range of motion and were provided with verbal encouragement.

Leg extension 1-repetition maximum strength testing was assessed in both legs separately using an incremental multiple repetition testing procedure with standard gym equipment (Life Fitness, Cambridge, UK) and was carried out individually for each leg with the leg randomized to be immobilized always being tested first as we have previously described (15). After two warm-up sets of eight and four repetitions at self-determined 25% and 50% of 1-RM, respectively, single repetitions at 1-RM were attempted. The weight was increased incrementally until no further weight could be lifted, with each attempt separated by a 2-min rest. The final 1-RM lift was taken as the heaviest repetition that was successfully completed with a correct technique where the full range of motion was achieved.

### Immobilization Protocol

Leg immobilization was administered using a unilateral leg brace (X-ACT Donjoy brace, DJO Global, Vista, CA) with the participant ambulating on crutches (after receiving instructions) for the duration of the immobilization period. The IMM leg was counterbalanced for leg dominance and the N-IMM leg acted as a within-participant control. The knee was fixed at an angle of 40° of flexion (with full knee extension being considered as 0°) by the locking hinge of the brace to ensure no weight-bearing occurred. Participants were instructed that all ground contact and muscle contractions of the IMM leg were forbidden (except for ankle rotation exercises twice per day to activate the venous pump). Adhesive tape with the experimenter’s signature inscribed was placed around the straps of the brace such that breaking of the tape would indicate tampering and result in exclusion from the study (9) (though this did not occur throughout the study). Participants were provided with a plastic cover to wear over the brace while showering. Daily contact was maintained with the participants to ensure compliance and any adjustments to the fitting of the brace were made solely by an experimenter.

### Diet

Participants’ habitual diets were recorded for 3 days (2 weekdays and 1 weekend day) before immobilization by a self-reported written diet diary following detailed instructions and advice from a member of the research team. Participants were asked to refrain from alcohol intake and maintain a similar diet throughout the immobilization period. This was assessed by further 2-day diet diaries completed during the first and last 2 days of immobilization, which were averaged to create an “immobilization diet.” Dietary analysis for the calculation of energy and macronutrient intake was completed using specific nutrition software (Nutritics; Swords, Co., Dublin, Ireland).

### Magnetic Resonance Imaging and Muscle Volume Calculations

A 1.5-tesla magnetic resonance imaging (MRI) scanner (Intera, Philips, The Netherlands) was used to obtain images of both thighs in the axial plane over the full length of the femur. A T1-weighted three-dimensional (3-D) turbo spin-echo sequence was used (field of view 500 × 500 mm, reconstructed matrix 512 × 512 mm, echo time 15 ms, repetition time 645 ms, slice thickness 5 mm, and slice gap 5 mm) with the subject laying still in the supine position, in line with our previous work (15). A 4-element send body radiofrequency (RF) coil was wrapped around both thighs. During the first scan, a specified distance from a bony landmark (femoral condyle) in the frontal plane was used to center the axial plane images. This distance was used on all subsequent scans to ensure that the axial images were in the same location along the length of the thigh. Slicer software (v. 4.10.0) (46) was used to analyze images obtained in the axial plane and calculate muscle volumes. First, the length of the femur between the lateral condyle and the greater trochanter was determined from the obtained images as previously described (47), and the top 25% of slices (from the greater trochanter working distally) and bottom 25% of slices (from the lateral condyle working proximally) were excluded so that only the middle 50% region of the length of the femur area was used for automated thresholding. The median number of slices was 22, which equaled a 22 cm midfemur region. The anatomical cross-sectional area (aCSA) of the thigh muscle on each slice was calculated by using the thresholding function in Slicer, with manual erasing applied as required to ensure only muscle tissue was thresholded (i.e., excluding bone, adipose tissue, and skin). Quadriceps muscle volume was determined by manually erasing the thresholding of nonquadriceps muscles (i.e., hamstring and adductor muscles) from the thresholding of thigh muscle on each slice. Nonquadriceps muscle volume was calculated by subtracting quadriceps muscle volume from thigh muscle volume. Muscle volume was calculated using a previously published method (47), where the total muscle cross-sectional area for all slices was calculated and multiplied by the slice thickness plus the distance between slices (linear interpolation) (in this case a total of 1 cm, 5-mm slice thickness, 5-mm slice gap), summarized by the following equation:

$$Muscle\;volume\;\left(cm^{3}\right)=\underset{aCSA}{\sum }\cdot (slice\;thickness+slice\;gap)$$

### Deuterated Water-Dosing Protocol

The deuterated water-dosing protocol was conducted in line with our previous work (16, 24) and consisted of a loading day on *day −4* of the experimental protocol followed by daily maintenance dosing on *days −3* to *1*. On *day −4*, participants arrived at the laboratory overnight fasted, and provided a background saliva sample. Then, participants ingested 400 mL of 70% deuterated water (70 atom percent; CK Isotopes Ltd., Leicestershire) separated into 50 mL aliquots ingested every 1.5 h. Participants remained in the laboratory until the fourth dose was consumed to monitor any side effects such as vertigo or dizziness (48, 49) (none were reported) with the remaining four doses being consumed at home under instruction on timings. To maintain body water deuterium enrichments of 0.8%–1.0% a daily maintenance dose of 50 mL was ingested upon waking on each day of the experimental period. Three hours after each maintenance dose, a saliva sample was collected by each participant at home using a cotton mouth swab (Celluron, Hartmann, Germany), which was lightly chewed for 1 min until saturated with saliva. The saturated swab was placed into an empty syringe and the saliva was dispensed into a collection tube and immediately frozen at home in the participant’s freezer. Frozen saliva samples were returned by participants on their next laboratory visit and stored at −80°C until subsequent analysis. To ensure uniformity and compliance with the deuterated water-dosing protocol and saliva collection, participants completed a daily log and returned empty containers and saliva samples at each laboratory visit.

### Body Water Deuterium Enrichment

Body water deuterium enrichment was measured in saliva samples on an automated online gas preparation system. A ThermoFisher Delta V Advantage isotope ratio mass spectrometer (IRMS; Bremen, Germany) equipped with a Finnigan GasBench II (ThermoFisher Scientific Waltham, MA) was used for stable hydrogen isotope ratio measurements. After uncapping a 12 mL Exetainer (Labco Limited, Lampeter, UK), 5 mg of activated charcoal and 200 mg of copper powder were introduced into the Exetainer followed by a platinum catalytic rod (all Thermo Fisher Scientific). The activated charcoal and copper powder were added to remove any potential contaminants in the samples that might poison the platinum catalyst. After putting 200 µL of saliva into the Exetainer, the Exetainer was recapped and placed into the GasBench II and flushed was a helium/hydrogen gas mixture. The samples were allowed to equilibrate for 4 h before the gas from the vial headspace was sampled by the automated analyzer and introduced into the IRMS for analysis. A standard calibration curve was prepared using 99.9% deuterium-enriched water (Sigma Aldrich, St. Louis, MO), and the deuterium (^2^H) enrichment in duplicate saliva samples was determined.

### Myofibrillar Bound [^2^H]Alanine Enrichments

The enrichments of [^2^H]alanine in the myofibrillar fraction of skeletal muscle tissue samples were determined as described previously (27). Briefly, ∼50 mg of whole frozen muscle was mechanically homogenized in 7.5 volumes of ice-cold homogenization buffer [50 mM Tris-HCL (pH 7.4), 1 mM EDTA, 1 mM EGTA, 10 mM β-glycerophosphate, 50 mM NaF, 0.5 mM activated sodium orthovanadate, and 1 complete mini protease inhibitor cocktail tablet per 50 mL of buffer (Roche Holding AG, Basel, Switzerland)]. Homogenized samples were centrifuged (2,200 *g* at 4°C for 10 min) and the pellet was washed in 500-µL of ice-cold homogenization buffer, centrifuged (700 *g* at 4°C for 10 min), and solubilized (750 µL of 0.3 M sodium hydroxide at 50°C for 30 min). After centrifugation (10,000 *g* at 4°C for 10 min), myofibrillar proteins were precipitated from the supernatant by adding 500 µL of 1 M perchloric acid and vortexing for 30 s and pelleted by centrifugation (700 *g* at 4°C for 10 min). The pellet was washed twice in 70% ethanol and amino acids were hydrolyzed in 2 mL of 6 M hydrochloric acid at 110°C for 24 h. The samples were subsequently dried under a vacuum (Savant SpeedVac, Thermo Fisher Scientific), reconstituted in 3 mL of 25% acetic acid, passed over cation exchange resin columns (100–200 mesh; H^+^ form; Dowex 50WX8; Sigma Aldrich Company Ltd., Gillingham, UK) and eluted with 6 M NH_4_OH, before being dried again under vacuum. Samples were resuspended in 1 mL distilled water and 1 mL of 0.1% formic acid in acetonitrile, centrifuged (10,000 *g* at 4°C for 3 min), and the supernatant was aliquoted, dried under a vacuum, and stored at −20°C. Amino acids were derivatized by adding 50 μL *N*-tert-butyldimethylsilyl-*N*-methyltrifluoroacetamide (MTBSTFA) + 1% tert-butyl-dimethylchlorosilane and 50 μL acetonitrile and vortexed and heated at 95°C for 40 min. The samples were then transferred to a gas chromatography vial. Alanine enrichment was analyzed using a ThermoFisher Delta V Advantage IRMS (Bremen, Germany) fitted with a Trace 1310 gas chromatograph with an online high-temperature thermal conversion oven (HTC) at 1,420°C. The sample (1 μL) was injected in splitless mode at an injection port temperature of 250°C. The peaks were resolved on a 30 m × 0.25 mm ID × 0.25 μm film Agilent Technologies DB-5 capillary column (temperature program: 110°C for 1 min; 10°C·min^−1^ ramp to 180°C; 5°C·min^−1^ ramp to 220°C; 20°C·min^−1^ ramp to 300°C; hold for 2 min) before pyrolysis. Helium was used as the carrier gas with a constant flow of 1 mL·min^−1^. Any amino acid eluting from the gas chromatograph was converted to H_2_ before entry into the IRMS. The enrichment of tracer was measured by monitoring ion masses 2 and 3 to determine the ^2^H/^1^H ratios of myofibrillar protein-bound [2H]alanine. A series of known standards were applied to assess the linearity of the mass spectrometer.

### Calculations

Myofibrillar protein fractional synthesis rates (FSR) were calculated based on the incorporation of [^2^H]alanine into myofibrillar protein with mean body water deuterium enrichment throughout the period in question being used as a surrogate for the true precursor pool (24, 41) using the standard precursor product equation:

$$FSR\;\left(\%\cdot day^{-1}\right)=\left[\frac{\Delta Ep}{E_{precursor}\;\times \;t}\right]\;\times \;100$$

where Δ*E*p represents the increment in [^2^H]alanine enrichment in myofibrillar protein between biopsies obtained on either *days 0* and *2*, *days 0* and *7*, or *days 2* and *7*; *E*_precursor_ represents the average body water deuterium enrichment between two biopsies corrected by a factor of 3.7 based upon the deuterium labeling of alanine during de novo synthesis (mean enrichment between *days 0* and *2*, *days 0* and *7*, or *days 2* and *7*); *t* represents the time between biopsies (*days 0* and *2*, *days 0* and *7*, or *days 2* and *7*).

### Statistical Analyses

To our knowledge, this is the first study dedicated to assessing the impact of muscle-damaging exercise on subsequent muscle deconditioning responses during disuse. As such, we performed a statistical power analysis by calculating an effect size based on the rates of muscle loss observed during muscle disuse due to limb fracture (i.e., “complicated disuse”) (50) compared with rates of muscle loss during an equivalent period of uncomplicated muscle disuse (51).

Differences in participant characteristics and the difference between groups in myofibrillar FSRs in the IMM leg corrected to the N-IMM leg between *days 0–7*, *0–2*, and *2–7* were analyzed using unpaired *t* tests. In line with our previous work investigating short-term uncomplicated muscle disuse (15, 24), statistical analyses were first performed using repeated-measures three-factor analysis of variance (ANOVA) tests [with condition (CON vs. DAM) as a between-participant factor, and time (0 vs. 2 vs. 7 days), and leg (IMM vs. N-IMM) as within-participant factors] to compare temporal differences in uncorrected (raw) muscle volumes, and uncorrected 1-RM (*days 0* and *7* only) in addition to myofibrillar bound [^2^H]alanine enrichments and myofibrillar FSRs (*days 0–2* vs. *days 2–7*). Subsequent two-factor leg (IMM vs. N-IMM) by time (0 vs. 2 vs. 7 days) ANOVA tests were used to investigate time *×* leg interactions, and group (CON vs. DAM) by time (0 vs. 2 vs. 7 days) ANOVA tests were used to investigate time *×* group interactions. Work from ourselves (26, 27) and others (52) has reported edematous swelling and a decline in muscle strength in response to eccentric exercise per se, which may act as extraneous variables as it relates to our primary hypotheses. As such, we also corrected muscle volumes, myofibrillar FSR, and 1-RM in the immobilized leg to the contralateral within-participant N-IMM ambulant leg at each time point (which also accounts for any potential changes in the muscle mass of the control leg due to alterations in habitual loading) and used repeated-measure two-factor ANOVA tests [with condition (CON vs. DAM) and time (0 vs. 2 vs. 7 days) as within-participant factors] to compare differences in “corrected” muscle volumes, myofibrillar FSR, and corrected 1-RM (*days 0–7* only). Body water deuterium enrichments and parameters of dietary intake (preimmobilization vs. immobilization diets) were also analyzed with the same two-factor ANOVA.

We have previously demonstrated a transient increase in daily free-living myofibrillar protein synthesis rates for up to 72 h after eccentric exercise (26, 27). Consequently, myofibrillar protein synthesis rates and muscle volumes may be regulated differently in response to eccentric exercise per se between *days 0* and *2* compared with *days 2–7* of immobilization. In view of this, we also repeated the statistical procedures applied to muscle volumes and myofibrillar protein synthesis rates detailed in the previous paragraph with the time factor representing a discreet immobilization period (i.e., *days 0–2* or *days 2–7* or *days 0–7*).

A Pearson’s correlation coefficient was used to assess the relationship between changes in quadriceps volume, myofibrillar FSRs, and 1-RM, and between changes in quadriceps volume and myofibrillar protein synthesis rates. For all ANOVAs, when a significant interaction effect was found, Šidák post hoc tests were applied to locate individual differences. Data are expressed as means ± standard error of the mean (SEM). Statistical significance was set at *P* < 0.05 and statistical analyses were performed with GraphPad Prism v. 9.0.1 (GraphPad Software, San Diego, CA).

## RESULTS

One participant in DAM withdrew on *day 2* of immobilization due to the inconvenience. Comparisons between CON and DAM, therefore, comprise *n* = 11 and *n* = 10, respectively, unless otherwise stated.

### Participant Characteristics and Diet

No differences in age, body mass, height, or BMI were detected between conditions (all *P* < 0.05; Table 1). Preimmobilization and immobilization diets’ energy and macronutrient (fat, protein, and carbohydrate) intakes did not differ within groups, nor were they different between groups (all *P* > 0.05; Table 2).

**Table 1.** Participants’ characteristics

	CON (*n* = 11)	DAM (*n* = 10)
Age, yr	20 ± 1	20 ± 1
Body mass, kg	74 ± 3	74 ± 5
Height, cm	178 ± 7	176 ± 2
BMI, kg·m^−2^	23 ± 1	24 ± 2

Values are represented as means ± SE. CON participants performed no eccentric contractions, whereas DAM participants performed 300 maximal eccentric muscle damaging knee extensor contractions in both legs immediately before the start of a 7-day unilateral leg immobilization period. BMI, body mass index. No significant differences were observed between groups (*P* > 0.05).

**Table 2.** Average dietary intake during a habitual period and during 1 wk of unilateral leg immobilization

	CON		DAM	
	Pre	During	Pre	During
Energy, MJ	11.0 ± 0.6	11.3 ± 3.0	12.0 ± 1.0	9.6 ± 1.1
Energy, Kcal	2,618 ± 153	2,689 ± 171	2,862 ± 237	2,283 ± 252
Protein, g·day^−1^	120 ± 6	111 ± 7	119 ± 13	98 ± 14
Protein, g·kgBM·day^−1^	1.7 ± 0.1	1.5 ± 0.1	1.7 ± 0.2	1.3 ± 0.1
Protein, %En	19 ± 1	17 ± 1	17 ± 1	18 ± 1
Carbohydrate, g·day^−1^	293 ± 23	311 ± 24	288 ± 29	254 ± 26
Carbohydrate, %En	45 ± 2	46 ± 2	41 ± 3	45 ± 3
Fat, g·day^−1^	103 ± 8	108 ± 8	123 ± 12	97 ± 11
Fat, %En	35 ± 2	36 ± 2	38 ± 2	36 ± 2

Values are represented as means ± SE. CON participants performed no eccentric contractions (*n* = 11), whereas DAM (*n* = 10) participants performed 300 maximal eccentric muscle damaging knee extensor contractions in both legs immediately prior to the start of a 7-day unilateral leg immobilization period. “Pre” represents average 3-day dietary intake before immobilization; “During” represents average dietary intake during the first and last 2 days of immobilization. No significant differences were observed within or between groups (*P* > 0.05).

### Skeletal Muscle Volumes

**Figure 2.**
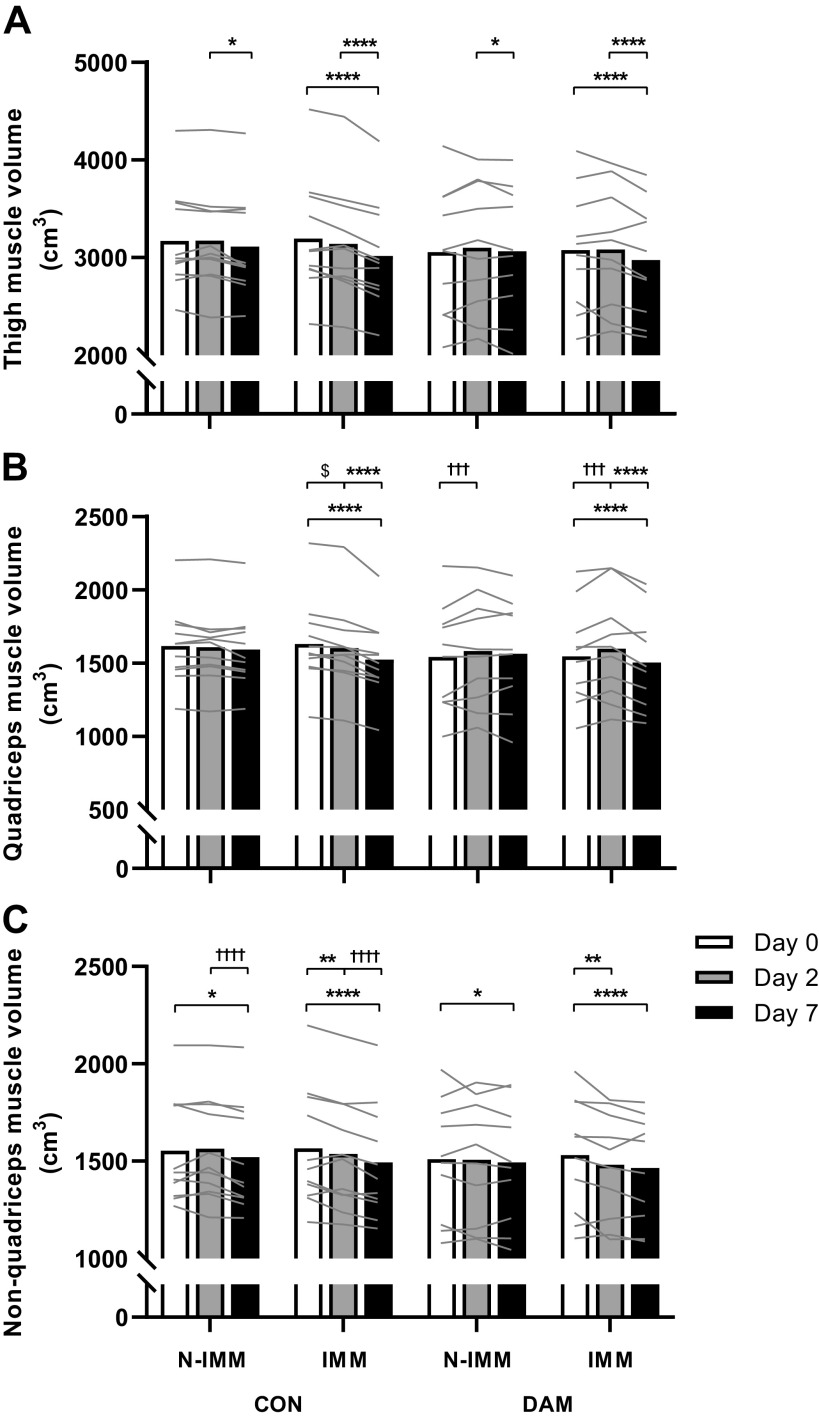
Thigh (*A*), quadriceps (*B*), and nonquadriceps (*C*) muscle volume determined using MRI at *day 0* and after 2 and 7 days of unilateral leg immobilization following either no exercise (CON; *n* = 11) or 300 eccentric muscle damaging knee extensor contractions performed in both legs immediately before immobilization (DAM; *n* = 10). Data are presented as means with lines representing individual responses. Statistical analysis was performed with separate three-factor ANOVAs. *Within leg difference between time points (i.e. time *×* leg interaction), †within leg difference between groups during corresponding immobilization period (i.e. time *×* group interaction), and $difference between time points in immobilized leg of control group only (i.e. three-way interaction). One symbol *P* < 0.05, two symbols *P* < 0.01, three symbols *P* < 0.001, and four symbols *P* < 0.0001.

Thigh, quadriceps, and nonquadriceps muscle volumes (Fig. 2) were not different between groups or between legs at *day 0* (all *P* > 0.05). When examining temporal changes in uncorrected muscle volumes (i.e., *day 0* vs. *day 2* vs. *day 7*), significant time *×* leg interactions (no three-way interactions; all *P* > 0.05) were detected for thigh (Fig. 2*A*), quadriceps (Fig. 2*B*), and nonquadriceps (Fig. 2*C*) muscle volumes (all *P* < 0.001). See Supplemental Table S1; https://doi.org/10.24378/exe.3543 for a complete list of all *P* values obtained from the analysis of skeletal muscle volumes.

Thigh muscle volume remained unchanged in the N-IMM leg (*P* > 0.05) but decreased in the IMM leg after 1 wk of immobilization (*P* < 0.001) in CON (by 5.4 ± 0.9%; from 3,195 ± 177 to 3,018 ± 162 cm^3^) and DAM (by 3.2 ± 1.5%; from 3,077 ± 194 to 2,975 ± 185 cm^3^) with no group interaction (*P* > 0.05). Temporally, this decrease was not evident after 2 days (*P* > 0.05) but was observed between *days 2* and *7* (*P* < 0.001) in CON (3.8 ± 0.5%) and DAM (3.4 ± 0.9%) with no group interaction (*P* > 0.05).

Quadriceps muscle volume remained unchanged in the N-IMM leg (*P* > 0.05) but decreased in the IMM leg, after 1 wk of immobilization (*P* < 0.001) in CON (by 6.4 ± 1.1%; from 1,630 ± 88 to 1,524 ± 79 cm^3^) and DAM (by 2.6 ± 1.8%; from 1,545 ± 105 to 1,504 ± 105 cm^3^) with no group interaction (*P* > 0.05). Temporally, this decrease was not evident after 2 days (*P* > 0.05) but was observed between *days 2* and *7* (*P* < 0.001) in CON (4.8 ± 0.8%) and DAM (5.8 ± 1.0%) with no group interaction (*P* > 0.05). A significant time *×* group interaction was also detected (*P* = 0.008) such that after 2 days quadriceps muscle volume did not change in CON but increased in DAM (i.e., in both legs irrespective of immobilization) (by 2.7 ± 1.6% from 1,542 ± 113 to 1,583 ± 117 cm^3^ in the N-IMM leg and by 3.4 ± 1.4% from 1,546 ± 105 to 1,599 ± 114 cm^3^ in the IMM leg; returning to baseline at 7 days).

Nonquadriceps muscle volume remained unchanged in the N-IMM leg between *days 0* and *7* (*P* > 0.05) but decreased temporally between *days 2* and *7* (*P* = 0.040) in CON (by 2.0 ± 0.7%; from 3,174 ± 152 to 3,114 ± 157 cm^3^) and DAM (by 1.1 ± 0.9%; from 3,099 ± 209 to 3,066 ± 207 cm^3^) with no group interaction (*P* > 0.05). In the IMM leg, nonquadriceps muscle volume decreased over 1 wk of immobilization (*P* < 0.001) in CON (by 4.5 ± 0.8%; 1,565 ± 91 to 1,494 ± 87 cm^3^) and DAM (by 4.1 ± 1.5%; from 1,531 ± 93 to 1,465 ± 85 cm^3^) with no group interaction (*P* > 0.05). Temporally, this decrease was evident after 2 days (*P* = 0.003) in CON (1.7 ± 1.0%) and DAM (3.0 ± 1.4%) with no group interaction (*P* > 0.05). Nonquadriceps muscle volume decreased further between *days 2* and *7* (*P* < 0.001) in CON (by 2.8 ± 0.6%) and DAM (by 1.1 ± 0.9%) with no group interaction (*P* > 0.05).

Given the regulation of muscle volume in response to prior eccentric exercise clearly appears different during the early (*days 0–2*) compared with later (*days 2–7*) immobilization periods, we also performed statistical analyses for each discreet immobilization period (i.e., *day 0* vs. *day 7*, *day 0* vs. *day 2*, and *day 2* vs. *day 7*). To that end, significant time *×* leg interactions were detected for thigh muscle volume (no three-way interactions; all *P* > 0.05) between *days 0* and *7*, *days 0* and *2*, and *days 2* and *7* (all *P* < 0.05; Fig. 2*A*). Specifically, thigh muscle volume remained unchanged in the N-IMM leg between *days 0* and *7* (*P* > 0.05) but decreased (*P* < 0.001) in the IMM leg CON and DAM with no group interaction (*P* > 0.05). Between *days 0* and *2* thigh muscle volume remained unchanged in the N-IMM and IMM legs of both groups (both *P* > 0.05). Between *days 2* and *7*, thigh muscle volume decreased modestly in the N-IMM leg (*P* = 0.014) in CON (by 2.0 ± 0.7%; from 3,174 ± 152 to 3,114 ± 157 cm^3^) and DAM (by 1.1 ± 0.9%; from 3,099 ± 209 to 3,066 ± 207 cm^3^) with no group interaction (*P* > 0.05), but decreased to a greater degree in the IMM leg (*P* < 0.001) in CON (by 3.8 ± 0.5%; from 3,140 ± 172 to 3,018 ± 162 cm^3^) and DAM (by 3.4 ± 0.9%; from 3,083 ± 194 to 2,975 ± 185 cm^3^) with no group interaction (*P* > 0.05).

Significant time *×* leg interactions were detected for quadriceps muscle volume between *days 0* and *7* and *days 2* and *7* (both *P* < 0.05; Fig. 2*B*). Specifically, between *days 0* and *7* and *days 2* and *7* quadriceps muscle volume remained unchanged in the N-IMM leg (both *P* > 0.05) but decreased in the IMM leg (both *P* < 0.001) in CON and DAM with no group interaction (*P* > 0.05). A significant three-way interaction (*P* = 0.020) was also detected for quadriceps muscle volume between *days 0* and *2* revealing that volume remained unchanged in the N-IMM leg of both groups (both *P* > 0.05) but decreased in the IMM leg of CON only (by 1.7 ± 0.5%; from 1,630 ± 88 to 1,602 ± 87 cm^3^; *P* = 0.031). Significant time *×* group interactions were also detected for quadriceps muscle volume revealing that, irrespective of immobilization, quadriceps volume decreased between *days 0* and *2* and *days 2* and *7* in CON (both *P* < 0.05) but did not change in DAM.

Significant time *×* leg interactions were detected for nonquadriceps muscle volume (no three-way interactions; all *P* > 0.05) between *days 0* and *7* and *days 0* and *2* (both *P* < 0.05; Fig. 2*C*). Specifically, nonquadriceps muscle volume decreased in the N-IMM leg (*P* = 0.041) and IMM leg (*P* < 0.001) between *days 0* and *7* in CON and DAM with no group interaction (*P* > 0.05). Between *days 0* and *2* nonquadriceps muscle volume did not change in the N-IMM leg (*P* > 0.05) but decreased in the IMM leg (*P* = 0.005) in CON and DAM with no group interaction (*P* > 0.05).

### Corrected Skeletal Muscle Volumes

**Figure 3.**
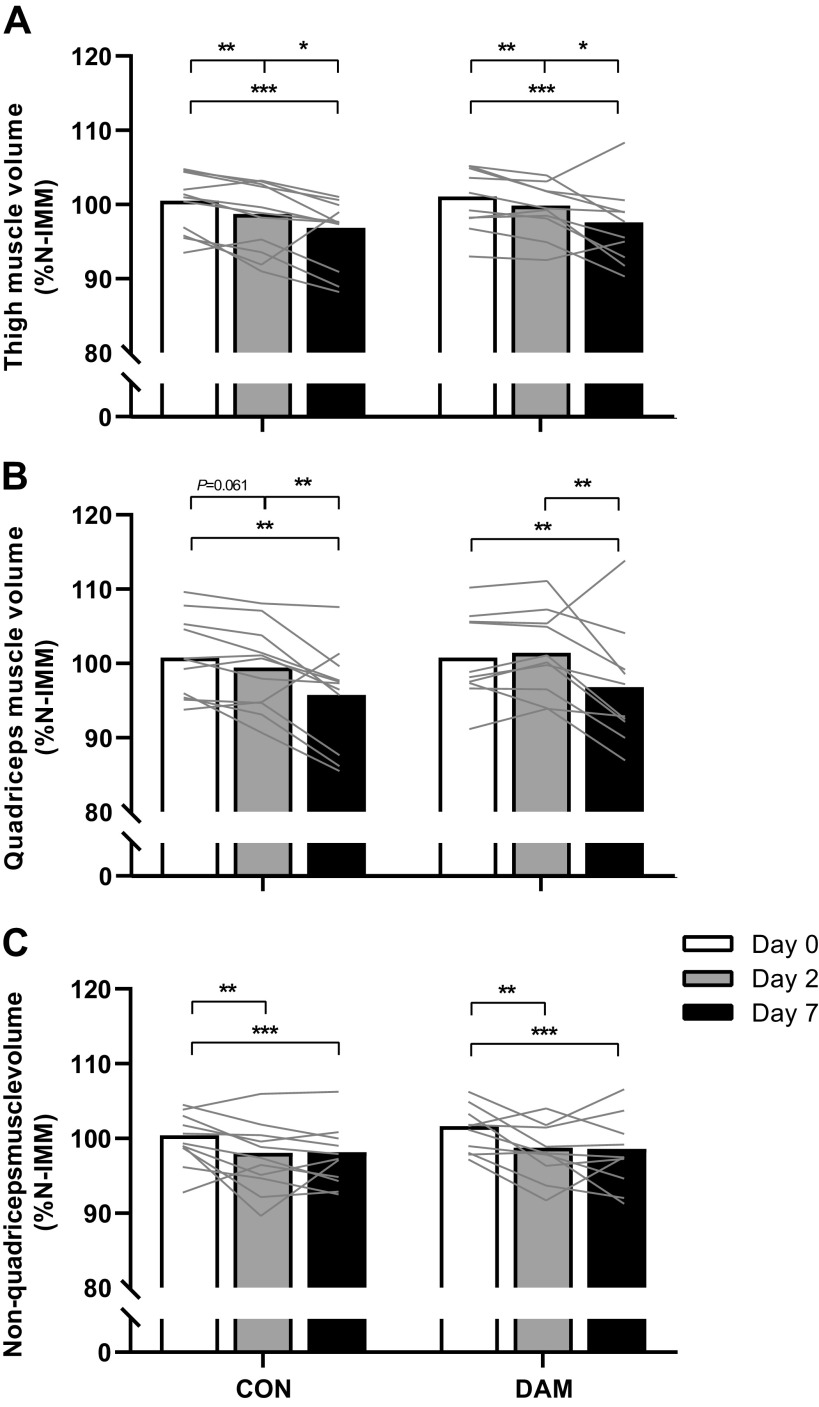
Thigh (*A*), quadriceps (*B*), and nonquadriceps (*C*) muscle volume in the immobilized leg corrected to the contralateral within-participant nonimmobilized leg at each time point. Muscle volumes were determined using MRI at *day 0* and after 2 and 7 days of unilateral leg immobilization following either no exercise (CON; *n* = 11) or 300 eccentric muscle damaging knee extensor contractions performed in both legs immediately before immobilization (DAM; *n* = 10). Data are presented as means with lines representing individual responses. Statistical analysis was performed with separate two-factor ANOVAs. *Difference between time points. One symbol *P* < 0.05, two symbols *P* < 0.01, and three symbols *P* < 0.001.

Thigh, quadriceps, and nonquadriceps muscle volumes of the IMM leg corrected (as a %) to the N-IMM leg (Fig. 3) were not different between groups at *day 0* (*P* > 0.05). Significant time effects (no time *×* condition interactions; all *P* > 0.05) were detected for thigh (*A*), quadriceps (*B*), and nonquadriceps (*C*) muscle volumes (all *P* < 0.001).

Thigh muscle volume decreased over the full week of immobilization (*P* < 0.001) in CON (by 3.7 ± 0.9%; from 100.5 ± 1.2 to 96.8 ± 1.4% N-IMM) and DAM (by 3.5 ± 1.2%; from 101.1 ± 1.3 to 97.6 ± 1.6% N-IMM) with no group interaction (*P* > 0.05). Temporally, this decrease was not evident after 2 days (*P* > 0.05) but was evident between *days 2* and *7* (*P* < 0.001) in CON (1.9 ± 1.0%) and DAM (2.3 ± 1.2%) with no group interaction (*P* > 0.05).

Quadriceps muscle volume decreased over the full week of immobilization in CON (by 5.0 ± 1.6%; from 100.8 ± 1.6 to 95.7 ± 2.0% N-IMM) and DAM (by 4.0 ± 1.9%; from 100.8 ± 1.8 to 96.8 ± 2.5% N-IMM) with no group interaction (*P* > 0.05). Temporally, this was not evident after 2 days (*P* > 0.05) but was evident between *days 2* and *7* (*P* < 0.001) in CON (3.7 ± 1.3%) and DAM (4.7 ± 1.8%) with no group interaction (*P* > 0.05).

Nonquadriceps muscle volume decreased over the full week of immobilization in CON (by 2.3 ± 0.8%; from 100.4 ± 1.0 to 98.1 ± 1.2% N-IMM) and DAM (by 2.9 ± 0.9; from 101.6 ± 1.0 to 98.6 ± 1.5% N-IMM) with no group interaction (*P* > 0.05). Temporally, this decrease was evident after 2 days (*P* = 0.002) in CON (2.3 ± 0.8%) and DAM (2.9 ± 1.1%) with no group interaction (*P* > 0.05) and no further decrease between *days 2* and *7* (*P* > 0.05).

We again performed additional analyses for each discreet immobilization period (i.e., *day 0* vs. *day 7*, *day 0* vs. *day 2,* and *day 2* vs. *day 7*). Thigh muscle volume decreased between *days 0* and *7*, *days 0* and *2*, and *days 2* and *7* (all *P* < 0.05) equivalently between CON and DAM (Fig. 3*A*). Quadriceps muscle volume decreased between *days 0* and *7* and *days 2* and *7* (both *P* < 0.05) in CON and DAM with no group interaction (*P* > 0.05) (Fig. 3*B*). In addition, a significant time *×* leg interaction was detected for quadriceps muscle volume between *days 0* and *2* such that quadriceps volume tended to decrease in CON (by 1.3 ± 0.6%; from 100.8 ± 1.6 to 99.4 ± 1.7% N-IMM; *P* = 0.061) but did not change in DAM (+0.6 ± 0.6%; from 100.8 ± 1.8 to 101.4 ± 1.8% N-IMM; *P* = 0.490). Nonquadriceps muscle volume decreased between *days 0* and *7* and *days 0* and *2* (both *P* < 0.05) in CON and DAM groups with no group interaction (*P* > 0.05) (Fig. 3*C*).

### Knee Extensor 1-Repetition Maximum

**Figure 4.**
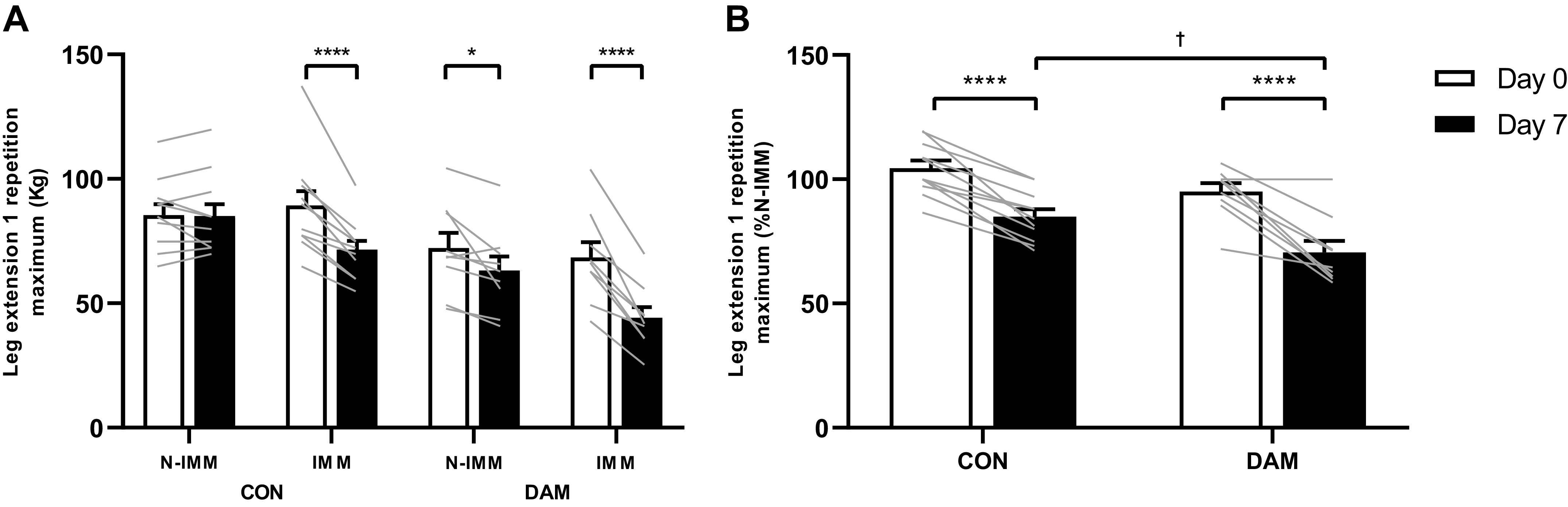
Leg extension 1-repetition maximum (1-RM) presented in both immobilized and nonimmobilized legs (*A*), and in the immobilized leg corrected to the contralateral within-participant nonimmobilized leg (*B*). 1-RM was determined before and immediately after 7 days of unilateral leg immobilization. Participants either performed no exercise (*n* = 11; CON) or performed 300 maximal unilateral eccentric muscle damaging quadriceps contractions in both legs (*n* = 9; DAM) immediately before immobilization. Data are presented as means with lines representing individual responses. Statistical analysis was performed with a three-factor (*A*) and two-factor (*B*) ANOVA. *Difference between time points, †difference between conditions on *day 7*. One symbol *P* < 0.05 and four symbols *P* < 0.0001.

Knee extensor 1-RM is presented for CON *n* = 11 and DAM *n* = 9 as one participant from DAM was unavailable to complete postimmobilization 1-RM testing. Knee extensor 1-RM was not different between CON and DAM at baseline (*P* > 0.05). A significant time *×* condition interaction was detected (with no three-way interaction; *P* > 0.05) in knee extensor 1-RM (*P* < 0.001; Fig. 4*A*). Specifically, knee extensor 1-RM did not change in the N-IMM leg of CON (from 85 ± 4 to 85 ± 5 kg; *P* > 0.05) but decreased in the IMM leg (by 19 ± 2%; from 89 ± 6 to 72 ± 3 kg; *P* < 0.001). However, in DAM knee extensor 1-RM decreased in the N-IMM (by 12 ± 4%; from 72 ± 6 to 63 ± 6 kg; *P* = 0.020) and IMM (by 35 ± 4%; from 68 ± 6 to 44 ± 4 kg; *P* < 0.001) legs. When correcting knee extensor 1-RM of the IMM leg as a percentage of the N-IMM leg, 1-RM decreased over time (*P* < 0.001) and exhibited a group difference (*P* = 0.014) such that there was no group difference at *day 0* (*P* > 0.05), but 1-RM was greater in CON compared with DAM at *day 7* (*P* = 0.013) (Fig. 4*B*).

### Body Water Deuterium and Myofibrillar Bound[^2^H]Alanine Enrichments

During sample analyses, it was clear that a participant in DAM had not disclosed their involvement in a recent study where a deuterium tracer had been administered, such that their background body water enrichment was an order of magnitude higher than the rest of the subject cohort. Due to such a clear outlier having the potential to confound the calculation of MyoPS rates, we chose to exclude this participant from all subsequent tracer related data (i.e., body water deuterium enrichments, myofibrillar bound [^2^H]alanine enrichments, and the subsequent calculations of myofibrillar fractional synthetic rates) which are therefore presented as CON; *n* = 11 and DAM; *n* = 9.

**Figure 5.**
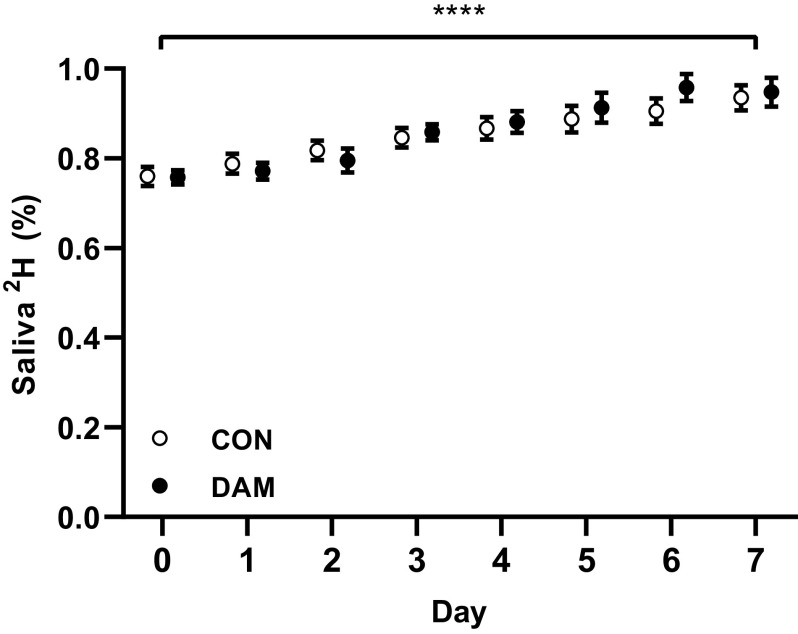
Daily **s**aliva deuterium enrichment. Participants either performed no exercise (*n* = 11; CON) or performed 300 maximal unilateral eccentric muscle damaging quadriceps contractions in both legs (*n* = 9; DAM) immediately before immobilization. Data are presented as means ± SE. Statistical analysis was performed with a two-factor ANOVA. *Difference between time points. Four symbols *P* < 0.0001.

Body water deuterium enrichments are presented in Fig. 5, and averaged 0.85 ± 0.01%, 0.78 ± 0.01%, and 0.88 ± 0.01% between *days 0* and *7*, *days 0* and *2*, and *days 2* and *7*, respectively. Body water deuterium enrichments showed a modest increase over time (*P* < 0.001) CON and DAM (*P* > 0.05) with no group interaction (*P* > 0.05).

Myofibrillar [^2^H]alanine enrichments [expressed as mole percent excess (MPE)] increased over time (*P* < 0.001) and a time *×* leg interaction effect was detected between *days 0* and *7* and *days 2* and *7* (*P* < 0.001). Between *days 0* and *7*, the increase in myofibrillar [^2^H]alanine enrichments was greater in the N-IMM compared with IMM leg and was equivalent in both CON (Δ0.376 ± 0.012 vs. Δ0.232 ± 0.017 MPE, respectively) and DAM (Δ0.482 ± 0.055 vs. Δ0.343 ± 0.046 MPE, respectively). Between *days 2* and *7* the increase in myofibrillar [^2^H]alanine enrichments was also greater in the N-IMM compared with IMM leg in both CON (Δ0.270 ± 0.014 vs. Δ0.154 ± 0.0145 MPE, respectively) and DAM (Δ0.281 ± 0.047 vs. Δ0.220 ± 0.042 MPE, respectively) with no group interaction (*P* > 0.05). Myofibrillar [^2^H]alanine enrichments between *days 0* and *7* and *days 2* and *7* were greater, irrespective of the leg, in DAM compared with CON (both *P* < 0.05).

### Daily Free-Living Myofibrillar Protein Synthesis Rates

**Figure 6.**
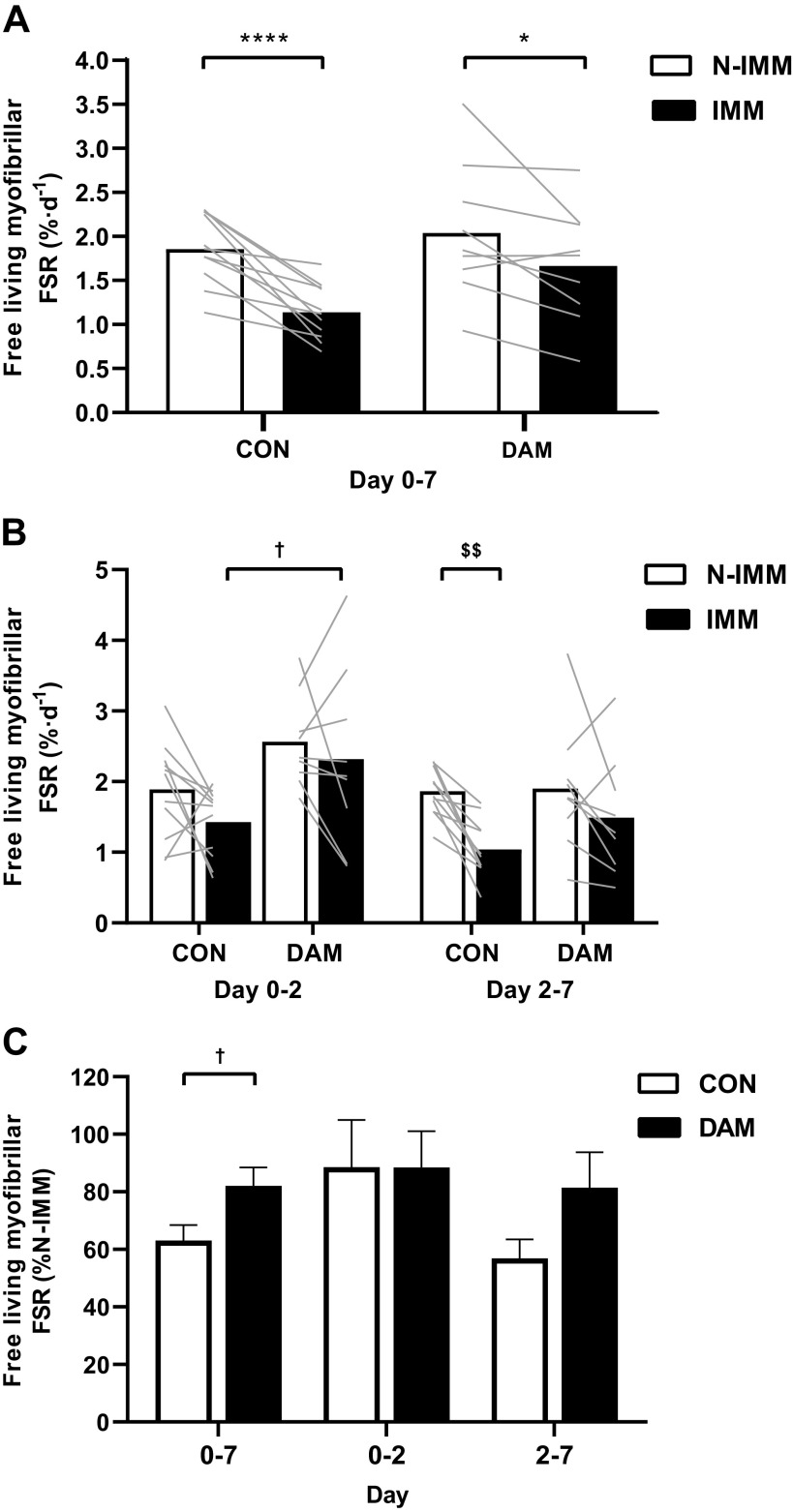
The average daily myofibrillar fractional synthetic rate (FSR) during the full 7-day immobilization period (*A*), during *days 0–2* and *days 2–7* (*B*) in nonimmobilized (N-IMM) and immobilized (IMM) legs and FSR in IMM corrected as a percentage of N-IMM (*C*). Participants either performed no exercise (*n* = 11; CON) or performed 300 maximal unilateral eccentric muscle damaging quadriceps contractions in both legs (*n* = 9; DAM) immediately before immobilization. Data are presented as means with lines representing individual responses and error bars representing SE (*C*). Statistical analysis was performed with separate two-factor ANOVAs. *Difference between time points, †difference between groups, $difference in FSR between legs in control group only during *days 2–7*. One symbol *P* < 0.05, two symbols *P* < 0.01 and four symbols *P* < 0.0001.

Average free-living daily myofibrillar FSRs are presented in Fig. 6. During the full 7-day immobilization period (*A*), daily myofibrillar FSRs were lower in the IMM compared with N-IMM leg (*P* < 0.001) in CON (by 37 ± 5%; 1.86 ± 0.11 vs. 1.14 ± 0.09%·day^−1^, respectively) and DAM (by 18 ± 6%; 2.04 ± 0.25 vs. 1.66 ± 0.22%·day^−1^, respectively) with no group interaction (*P* > 0.05).

When examining temporal changes in daily myofibrillar FSRs from *days 0* and *2* to *days 2* and *7* (*B*), effects of time, group, and leg were all detected (all *P* < 0.05, no two-way or three-way interactions; all *P* > 0.05). Specifically, myofibrillar FSRs were lower between *days 2* and *7* compared with *days 0* and *2* (i.e., irrespective of leg or group), were greater in DAM compared with CON (i.e., irrespective of leg or time) and lower in the IMM compared with N-IMM legs (i.e., irrespective of group or time). To determine what was driving these main effects, we performed additional statistical analyses of the discreet immobilization periods (i.e., *days 0* and *2* and *days 2* and *7*). Between *days 0* and *2* of immobilization, DAM exhibited higher myofibrillar FSRs compared with CON (*P* = 0.015), which was most profound in the IMM leg (48% greater; 2.57 ± 0.22 vs. 1.89 ± 0.21%·day^−1^, respectively; *P* = 0.035). Between *days 2* and *7* of immobilization, myofibrillar FSRs were lower in the IMM compared with N-IMM leg (*P* < 0.001); however, this was only observed in CON (by 43 ± 7%; 1.86 ± 0.10 vs. 1.04 ± 0.12%·day^−1^, respectively; *P* = 0.002) and not DAM (*P* > 0.05). Daily myofibrillar FSRs in the IMM leg after correcting to the N-IMM leg was greater in DAM compared with CON between *days 0* and *7* (*P* = 0.035) and also tended to be greater in DAM compared with CON between *days 2* and *7* (*P* = 0.079) (Fig. 6*C*).

### Correlations

**Figure 7.**
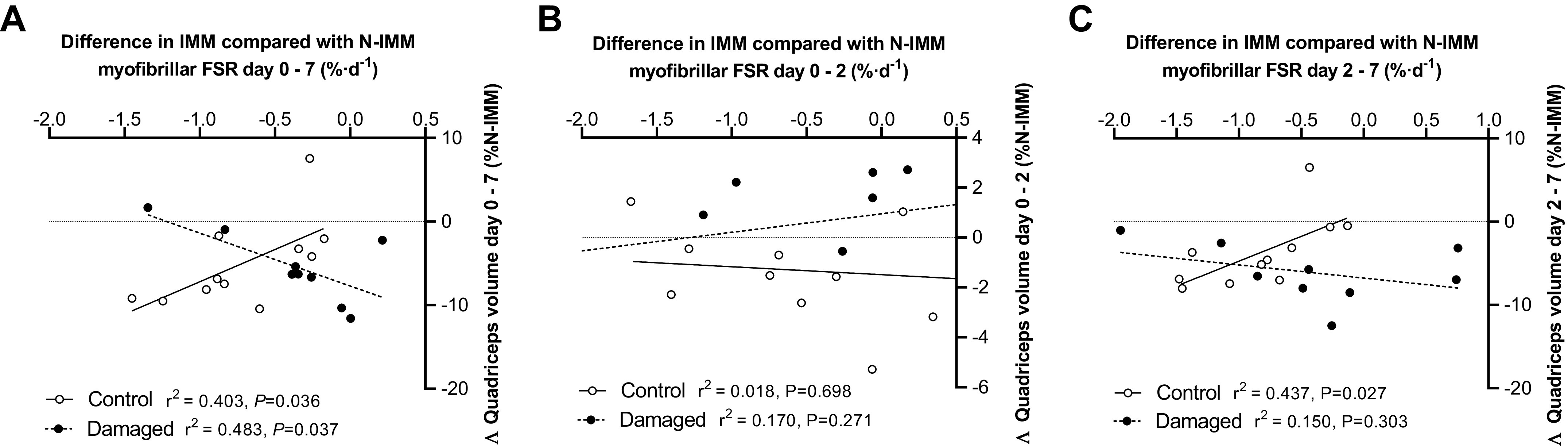
Correlations between the change in quadriceps volume in the immobilized leg and the difference in the daily myofibrillar FSR between the nonimmobilized and immobilized leg during the full 7-day immobilization period (*A*), and during *days 0–2* (*B*), and *days 2–7* (*C*). Participants either performed no exercise (*n* = 11; CON) or performed 300 maximal unilateral eccentric muscle damaging quadriceps contractions in both legs (*n* = 9; DAM) immediately before immobilization. Statistical analysis was performed with a Pearson’s correlation and *r*^2^ and *P* values are displayed on each graph. FSR, fractional synthetic rate.

**Figure 8.**
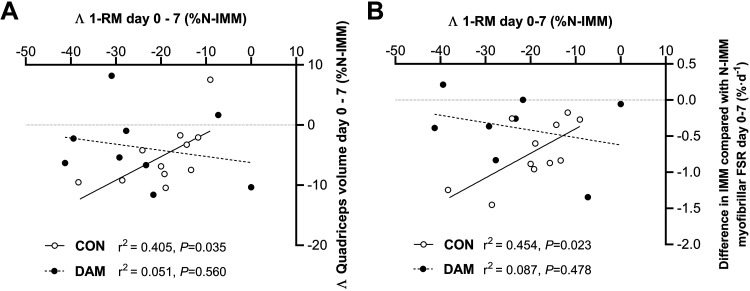
Correlations between the change in corrected quadriceps volume and the change in corrected knee extensor 1-repetition maximum (1-RM) (*A*), and between the change in daily myofibrillar FSRs and the change in corrected knee-extensor 1-RM (*B*) in the immobilized leg during the full 7-day immobilization period. Participants either performed no exercise (*n* = 11; CON) or performed 300 maximal unilateral eccentric muscle damaging quadriceps contractions in both legs [*n* = 9 (*A*), *n* = 8 (*B*); DAM] immediately before immobilization. Statistical analysis was performed with Pearson’s correlations and *r*^2^ and *P* values are displayed on the graph. FSR, fractional synthetic rate.

The difference in daily myofibrillar FSRs between IMM and N-IMM legs (as a single value representing the magnitude of impact of disuse on MPS for each individual) positively correlated with the magnitude of decrease in corrected quadriceps volume over the full week of immobilization (*r*^2^ = 0.403, *P* = 0.036; Fig. 7*A*) and between *days 2* and *7* of immobilization (*r*^2^ = 0.437, *P* = 0.027; Fig. 7*C*) in CON. Conversely, the difference in daily myofibrillar FSRs between the N-IMM and IMM leg correlated negatively with the change in corrected quadriceps volume over the full week of immobilization in DAM (*r*^2^ = 0.483, *P* = 0.037; Fig. 7*A*). Similarly, the magnitude of decrease in knee extensor 1-RM positively correlated with the magnitude of decrease in corrected quadriceps volume after 7 days of immobilization in the CON (*r*^2^ = 0.0405, *P* = 0.035) but not DAM (*r*^2^ = 0.051, *P* = 0.560) (Fig. 8).

## DISCUSSION

In the present work, we assessed the temporal modulation of short-term (2 and 7 days) “uncomplicated” muscle disuse deconditioning by the presence of prior muscle damage in young healthy males; a novel paradigm to experimentally model more clinically relevant “complicated” muscle disuse. The principle novel findings of this work were fourfold: *1*) 300 maximal muscle-damaging eccentric quadriceps contractions performed immediately before 1 wk of unilateral knee immobilization increased myofibrillar protein synthesis (MyoPS) rates and prevented disuse-induced loss of muscle volume after 2 days; *2*) This effect was transient, as evidenced by declining muscle volumes during the subsequent 5 days of immobilization (i.e., *days 2–7*) being unaffected by prior damaging exercise, despite exercise preventing the immobilization-induced decline in MyoPS rates; *3*) The tight positive correlation of declines in MyoPS rates and muscle volume during uncomplicated immobilization was inverted in damaged immobilized muscle; and *4*) Prior muscle damage exacerbated the decline in quadriceps strength caused by 7 days of immobilization (indeed confirming the damage incurred) and removed the correlations (observed with uncomplicated disuse) between the loss of muscle volumes and function, and decline in MyoPS rates and function.

“Uncomplicated” muscle disuse refers to the withdrawal of muscle contraction per se without the concomitant presence of clinically relevant factors (either systemic or intrinsic to the muscle) that may be expected to influence muscle atrophy (53). In the present work, we modeled (in our control group) uncomplicated muscle disuse atrophy using the established within-participant unilateral knee immobilization versus ambulant leg approach and replicated previous findings (2, 3, 6, 15, 24, 54). We report, relative to the contralateral ambulant leg, a 1.8% (or 0.9% per day) and 3.7% (or 0.5% per day) loss of thigh muscle volume after 2 and 7 days of immobilization, respectively (see Fig. 3). This likely translates to ∼57 g and ∼184 g of total muscle tissue lost from the analyzed region of the immobilized thigh (i.e., the central 50% portion of the femur) of which the majority (51% and 60%) was accounted for by quadriceps atrophy (∼29 g and ∼111 g muscle tissue) after 2 and 7 days, respectively (15). However, the burden of disuse in sports or clinical settings (i.e., due to injury or illness) is complicated by several accompanying physiological processes all known to influence the regulation of muscle mass; for example, systemic and local inflammation (29, 30), contraction-induced changes in muscle protein turnover (55), muscle damage and consequent cytokine release (26, 27), hypercortisolaemia (56), hypoxia (57), and edema (10). We (26, 27) and others (35, 36, 58) have shown that a single bout of high-volume and high-intensity eccentric exercise causes muscle damage, which leads to increases in daily MyoPS rates for 72 h, muscle and systemic inflammation, and edema. Eccentric exercise-induced muscle damage is, therefore, an ideal model with which to investigate the surplus effects of such clinically relevant processes on the rate of muscle disuse atrophy above that of disuse alone. Here, we report eccentric exercise-induced muscle damage (specifically of the quadriceps) immediately before limb immobilization transiently prevented early quadriceps muscle atrophy both when examining absolute changes in muscle volume (see Fig. 2) and when correcting between immobilized and nonimmobilized legs to account for damaged-induced edematous swelling of both legs and changes in volume to the control leg (see Fig. 3). Interestingly, however, this effect was restricted to the quadriceps muscle only (i.e., no effect on nonquadriceps muscle; suggesting local and intrinsic regulatory mechanisms) and did not persist between 2 and 7 days of immobilization, such that muscle loss during this period was comparable between conditions for all muscle groups. The lack of effect in this latter period seemingly “diluted” the early protective effect such that quadriceps muscle volume over the entire week of immobilization was also unaffected by prior damaging exercise.

Mechanistically, it has been established that rapid declines in postabsorptive (9, 22) and postprandial (3, 7, 9), and resultantly daily (5, 16, 24), MyoPS rates primarily drive uncomplicated muscle disuse atrophy in humans. Here, we used a deuterated water stable isotope approach (Figs. 5 and 6) to measure temporal in vivo and free-living daily MyoPS rates during immobilization of healthy volunteers in the presence or absence of muscle damage. In support of the key role daily MyoPS rates play in regulating muscle mass during disuse, we observed a 37% decline in the immobilized leg compared with ambulant leg in our control condition over the 7-day immobilization period (see Fig. 6). This effect manifested primarily over the later phase of immobilization and likely accounted for at least 58% of total observed quadriceps muscle loss (24). In line with the causal link, the individual magnitude of decline in daily MyoPS rates positively correlated with the extent of quadriceps volume lost between *days 0* and *7* and *days 2* and *7* (see Fig. 7). However, in keeping with our previous work (26, 27), the execution of damaging eccentric exercise increased MyoPS rates between 0 and 2 days in both the immobilized and nonimmobilized legs (see Fig. 6) and to an extent where the immobilized and nonimmobilized were expressing rates considerably higher (45% and 30%, respectively) than the control group. As such, eccentric exercise-induced muscle damage stimulates an increase in daily MyoPS rates for at least 2 days irrespective of that tissue being subjected to disuse or ambulation. This stimulation of MyoPS rates may even be more potent (or delayed) in an immobilized compared with ambulant leg evidenced by the ability of eccentric contractions to prevent the disuse-induced decline in MyoPS rates for at least 7 days. Indeed, increases in daily MyoPS rates across legs in the damaged condition provide compelling evidence that intense muscle contraction overcame any potential interfering effects of damage and inflammation on the regulation of muscle protein synthesis, at least over 1 wk (29, 30, 59–61). Damage-induced edematous swelling in the nonimmobilized leg was evident after 2 but not 7 days, and we have assumed that temporal changes in postexercise edematous swelling occur comparably between the immobilized and nonimmobilized legs. Given prevention of muscle disuse atrophy consequent to quadriceps muscle damage over the first 2 days of disuse is temporally aligned with increased daily MyoPS rates, this leads us to conclude that increased daily MyoPS rates, rather than changes in fluid shifts, mechanistically explains the prevention of muscle disuse atrophy during this early immobilization phase. Further work is clearly warranted to establish if this effect can be applied to multiple muscle groups (i.e., hamstrings), recreated [more (or less) effectively] with other modalities of exercise not involving damage (e.g., concentric resistance exercise, aerobic exercise, etc.) and to establish if the same effect is observed when factors that can complicate muscle disuse (i.e., inflammation, muscle damage, etc.) are present in different severities and without a possible protective effect from prior muscular contraction. Indeed, the latter will be key when translating this mechanistic model to more clinically relevant scenarios. However, worthy of note, recent work shows that 4 bouts of traditional concentric resistance leg exercise performed during the week before 5 days of bed rest did not attenuate declines in MyoPS rates or muscle loss in older men (62), suggesting contraction modality and/or damage per se may be crucial.

An additional striking observation was that complicated muscle disuse dissociates (at least to some extent) the apparent regulatory link we observed with uncomplicated disuse between MyoPS rates and muscle atrophy during the latter immobilization phase (i.e., *days 2–7*) and inverts the associations of muscle volumes with MyoPS rates over the full week (see Fig. 7). Indeed, we observed that although eccentric exercise statistically prevented the immobilization-induced decline in MyoPS rates during the latter immobilization phase and attenuated the decline in MyoPS rates during the whole week of immobilization after correcting to the nonimmobilized leg (see Fig. 6*C*), muscle volumes continued to decline in unison with uncomplicated disuse. This leads us to propose that, at least, in part, MyoPS-independent regulation of muscle disuse atrophy may occur in the presence of muscle damage, particularly once the early anabolic effect of maximal muscle contraction has subsided. Minimal direct metabolic measurements concerning the role of muscle protein breakdown on human muscle disuse atrophy or recovery from muscle damage are available. Static indirect data suggest that uncomplicated muscle disuse (2, 5, 6, 11, 24, 63) and muscle damage (64, 65) initiate molecular processes associated with multiple proteolytic pathways. It could be argued that in the present investigation immobilization combined with muscle damage increased muscle protein breakdown yielding an abundance of free amino acids sufficient to stimulate MyoPS rates, as reported in other inflammatory states (66), albeit maintaining a negative protein balance. Consequently, it is intriguing to speculate if our present data display equivalent rates of muscle loss between groups, but arrived at by considerably different mechanisms.

As in previous work (15), we observed that the 3.7% loss of quadriceps muscle volume across 1 wk of immobilization in our control group was associated (and correlated; see Fig. 8) with (a 19%) loss of quadriceps muscle strength, with the ambulant leg unaffected (see Fig. 4). Reassuringly, in the damaged group, we observed a loss of quadriceps muscle strength in both legs showing the characteristic (35) long(er)-term presence of muscle damage per se. However, this effect was exacerbated in the immobilized leg (both relative to the within-participant ambulatory leg and the corresponding leg of the control group). Therefore, despite providing an early (i.e., over 2 days) protective effect against muscle disuse atrophy, prior muscle damage clearly impairs the ability to recuperate disuse-induced loss of muscle strength over one week of immobilization, independently of the decline in muscle volume (see Fig. 8*A*). Moreover, the significant negative correlation between the individual decline in MyoPS rates and 1-RM in the control group was not observed with prior eccentric exercise (see Fig. 8*B*). This extends on our recent work where we reported that daily changes in muscle contractile function after muscle damage occur without changes in MyoPS rates and instead proposed elevated muscle protein breakdown as a potentially important process (26, 27). This is of important applied relevance given the likelihood that disuse will often be undertaken while recuperating from musculoskeletal injury (1) and lends further support to our conclusions that the presence of muscle damage disassociates the regulatory link between MyoPS rates, muscle mass, and muscle function that is present during uncomplicated disuse.

In conclusion, we demonstrate that the rapid decline in daily MyoPS rates induced by short-term limb immobilization of healthy volunteers is attenuated by the prior execution of a single bout of muscle-damaging eccentric contractions, and this is associated with a transient (after 2, but not 7 days) prevention of quadriceps muscle atrophy. Presence of muscle damage appears to add complexity to the physiological mechanisms responsible for uncomplicated muscle disuse atrophy evidenced by the removal of the correlations between changes in MyoPS rates, muscle volume, and muscle function. These novel data highlight the relevance of considering the application of more “complicated” models of in vivo muscle disuse atrophy to study both applied and mechanistic outcomes.

## SUPPLEMENTAL DATA

Supplemental Fig. S1: https://doi.org/10.24378/exe.3543.

## GRANTS

This work was part of a PhD studentship grant supported by the University of Exeter (to F.B.S.). A.J.M. and D.R.A. are supported in part by a grant from the National Institute of Aging (P30-AG024832).

## DISCLOSURES

No conflicts of interest, financial or otherwise, are declared by the authors.

## AUTHOR CONTRIBUTIONS

T.S.O.J., F.B.S., and B.T.W. conceived and designed research; T.S.O.J., S.P.K., J.F., M.L.D., and B.T.W. performed experiments; T.S.O.J., D.R.A., A.J.M., and B.T.W. analyzed data; T.S.O.J., F.B.S., and B.T.W. interpreted results of experiments; T.S.O.J. prepared figures; T.S.O.J., F.B.S., and B.T.W. drafted manuscript; T.S.O.J., M.L.D., F.B.S., and B.T.W. edited and revised manuscript; T.S.O.J., S.P.K., J.F., D.R.A., A.J.M., M.L.D., F.B.S., and B.T.W. approved final version of manuscript.
